# Analyzing the metabolic fate of oral administration drugs: A review and state-of-the-art roadmap

**DOI:** 10.3389/fphar.2022.962718

**Published:** 2022-10-07

**Authors:** Liu Liu, Yiming Liu, Xiaojie Zhou, Zhiwei Xu, Yehui Zhang, Liting Ji, Chunlan Hong, Changyu Li

**Affiliations:** School of Pharmaceutical Sciences, Zhejiang Chinese Medical University, Hangzhou, China

**Keywords:** oral drugs, metabolism pathway, metabolism enzymes, metabolic models, model application

## Abstract

The key orally delivered drug metabolism processes are reviewed to aid the assessment of the current *in vivo/vitro* experimental systems applicability for evaluating drug metabolism and the interaction potential. Orally administration is the most commonly used state-of-the-art road for drug delivery due to its ease of administration, high patient compliance and cost-effectiveness. Roles of gut metabolic enzymes and microbiota in drug metabolism and absorption suggest that the gut is an important site for drug metabolism, while the liver has long been recognized as the principal organ responsible for drugs or other substances metabolism. In this contribution, we explore various experimental models from their development to the application for studying oral drugs metabolism of and summarized advantages and disadvantages. Undoubtedly, understanding the possible metabolic mechanism of drugs *in vivo* and evaluating the procedure with relevant models is of great significance for screening potential clinical drugs. With the increasing popularity and prevalence of orally delivered drugs, sophisticated experimental models with higher predictive capacity for the metabolism of oral drugs used in current preclinical studies will be needed. Collectively, the review seeks to provide a comprehensive roadmap for researchers in related fields.

## Introduction

Globally, oral administration is still the way for most clinical drugs to delivery. Despite the rapid development of intravenous, subcutaneous or intramuscular injection, oral drug delivery is still considered to be the preferred route in terms of good compliance and ease of administration (McConville, 2017; Manconi et al., 2020). In fact, professionals in the pharmaceutics field have made many new attempts in oral administration routes and doses to increase the drug efficacy in recent years (Hens et al., 2018; Jash et al., 2021). The successful discovery and development of drug candidates include evaluating the fluctuation of indicators *in vivo* to predict its effectiveness after being delivered to the intended site. The fate of drugs is supposed to be absorbed or excreted through various pathways after entering the living. That is, the life of a drug is being invented and created, then disappears after completing the treatment. Drugs experience absorption, distribution, metabolism and excretion (ADME) while completing their therapeutic errand (Szakács et al., 2008). The term, *drug metabolism*, refers to the metabolic process in which the parent compound is converted into metabolites to facilitate elimination, also known as bioconversion. The metabolism of drugs after oral administration can be roughly divided into oxidation, reduction, hydrolysis and conjugation. Among them, oxidation, reduction and hydrolysis belong to phase I metabolism, and conjugation belongs to phase II metabolism (Yengi et al., 2007). There are two results of drug bioconversion under a variety of drug-metabolizing enzymes (especially liver enzymes) action. On the one hand, it becomes a drug with no pharmacological activity (inactivation). On the other hand, the non-pharmacologically active substances are converted into pharmacologically active metabolites, e.g., prodrug, and even toxic metabolites are produced (active activation). By definition, prodrugs are derivatives or precursors of therapeutically active molecules, which undergo bioconversion into their active form inside the body, be it *via* spontaneous processes (e.g., hydrolytic degradation) or through a biocatalytic mechanism.

The drug bioconversion depends on their peculiar physicochemical properties and the interaction between the drug carrier and different sites (Liu et al., 2021). Emphasis routinely has been placed on the liver due to the presence of all organelles and their associated drug-metabolizing enzymes (Underhill and Khetani, 2018). Most clinical drugs phase I and phase II metabolic reactions occur with the participation of the liver drug-enzyme system. It can be seen that the liver plays an irreplaceable role in metabolism process (Alves-Bezerra and Cohen, 2017; Xu et al., 2020). The metabolism and elimination of drugs in the gastrointestinal tract (GIT) is a complex and dynamic process involving many mechanisms and pathways. GIT not only affects the metabolism of drugs, but also affects the absorption and transport due to the presence of multiple transporters (Shugarts and Benet, 2009). Flora has gradually attracted attention *via* participate in drug metabolism by degradation, hydrolysis and reduction in recent years (Clarke et al., 2014). Assuredly, phase I drug-metabolizing enzymes (CYP2B6, CYP2C8, CYP2C9, CYP2C19, CYP2D6, CYP3A4, CYP3A5) and phase Ⅱ drug-metabolizing enzymes (UGTs, SULTs) are known to be expressed in the human intestine (Li et al., 2020). These enzymes are able to participate in a wide variety metabolism process *via* oxidation, glucuronidation reaction and sulfonation reaction, *etc.* Metabolism in the liver and the GIT is the important determinants of the overall disposition of drugs, and metabolites formed can have an impact on the efficacy and safety of humans. Comprehend the factors and physiological barriers that influence drug metabolism and bioconversion is necessary to develop drug candidates with optimal therapeutic efficacy.

In order to the development of the pharmaceutical industry, an in-depth comprehension and application of the hidden mechanisms and different factors involved in drug metabolism at different stages of drug development, as well as good predictive models are required. Over the last few decades, *in vivo/vitro* models are being gradually developed to study the delivery procedure of drug molecules after oral administration. Considering genetic polymorphisms as well as the environment, diet and lifestyle vary widely among human subjects, human beings are generally used only for the clinical validation phase of the final marker. The metabolic pathways of cells are highly conserved among species and not universal, however, the advantages of cell models include low drug dosage, low cost, high speed, and suitability for high-throughput screening. Furthermore, it is relatively easy to obtain animals various biological samples, and the administrative treatment is highly operable. The animal experiments conditions are easy to control, and individual differences in animals are less variable compared to human beings. Understanding the metabolic mechanisms of drug candidates using metabolic models greatly aids in the development of drug delivery systems with optimal properties. This article focuses on the mechanism of drug metabolism and different experimental models. It is possible to simulate the *in vivo* environment by culturing cells in related parts *in vitro* to explore drugs metabolic procedure. In addition, there are experimental bacterial bioconversion models, microsomes, *ex vivo* tissue section, and animal models with editing genes for different experimental purposes. Herein, we evaluated the strengths and limitations of altered metabolism models. These models may have significant potential to exploit in the preclinical drugs screening.

## Drug metabolism process

**FIGURE 1:**
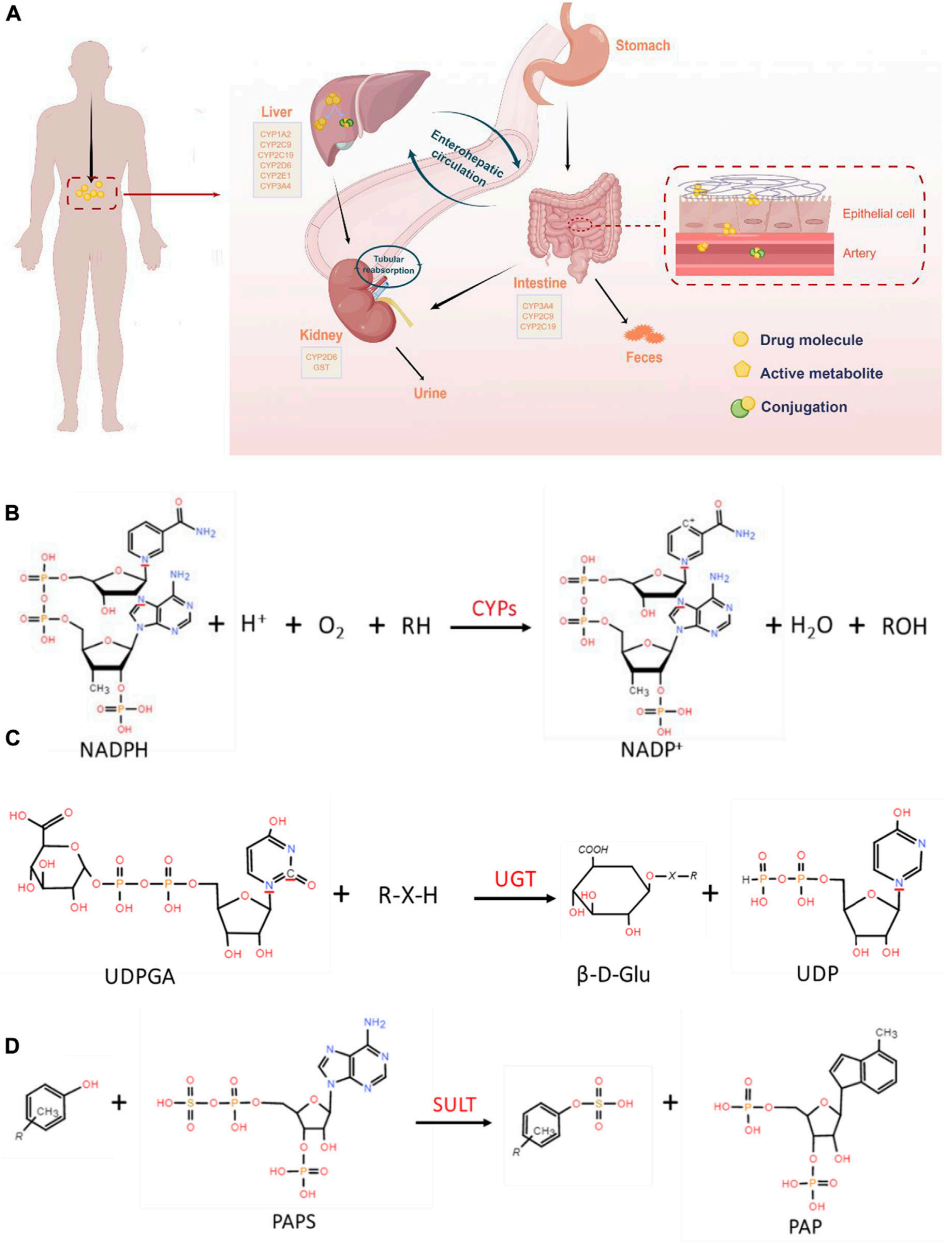
**(A)**. Schematic overview of the drug metabolic process *in vivo* (By Figdraw, www.figdraw.com). Oral drugs are metabolized by metabolic enzymes after entering the GIT and liver, and some metabolites are excreted by the kidney. **(B)**. General reaction catalyzed by CYP450. **(C)**. Conjugation of a nucleophile substrate with UDPGA catalyzed by UGTs. **(D)**. Metabolic reactions involved in SULT.

The natural assimilation process of an orally administered drug involves the breakdown of its components, which are then metabolized primarily by the GIT and the liver due to high levels of metabolic enzymes exists. The metabolism of drugs in the GIT and liver is a complex process, as illustrated in Figure 1. Generally, drugs experienced complex metabolic reactions under various drug-metabolizing enzymes (especially hepatic drug-metabolizing enzymes) and transporters action. In most cases, the polarity of drug metabolites is greater than that of the original drug to facilitate excretion. But there is also the opposite metabolism, such as the acetylated sulfonamides (Marshall, 1954; Shear et al., 1986) or the methylated phenolic hydroxy (Zhang et al., 2020). Interestingly, some drugs are not completely metabolized or some metabolites are still excreted in the original form after many complex steps (Cai et al., 2022). Drugs have distinct destinies due to different physicochemical properties, including inactivation, activity decreased (Brandao, 1977), activity enhanced (Molet et al., 1997), activation (B'Hymer and Cheever, 2010) and toxic metabolites production (Brune et al., 2015). The metabolism of drugs is closely related to their efficacy and safety. Reducing or even avoiding the toxic and side effects can be achieved by studying drugs metabolism properties and laws to improve the bioavailability and efficacy of drugs.

### Phase I metabolism pathway

A series of reactions of most drugs occurs under the specific enzymes catalyze, which lead to structure and physicochemical properties changed (Iyanagi, 2007). Drug metabolism is the major source of pharmacokinetic variability in human beings. At the root of this changeability are the phenotypic as well genotypic differences in the expression of the enzymes involved in the metabolism of drugs (Callegari et al., 2013; Zhao et al., 2014). Nicotinamide adenine dinucleotide phosphate (NADPH) -cytochrome P450 (CYP450) is one of the most common phases I drug-metabolizing enzymes that require NADPH as a cofactor (Hurst et al., 2007; Iyanagi, 2007). CYPs, also called hydroxylase and mixed function oxidase (MFO), catalyzes the incorporation of O in the O_2_ with lipid-soluble substrates to form hydroxylates or epoxides, and the other O is reduced to H_2_O by NADPH. The reaction formula is illustrated in Figure 1B. It plays an extremely important role in the exogenous and endogenous substances metabolism process (Song et al., 2021). Human hepatocyte CYPs are divided into five major families, including CYP1, CYP2, CYP3, CYP7 and CYP27, among which CYP1, CYP2, and CYP3 are mainly involved in heterologous substances bioconversion (Sarlis and Gourgiotis, 2005). Different CYP families are divided into A, B, C and other sub-families according to amino acid sequence homology. Among them, CYP3A4, CYP2C9, CYP1A2 and CYP2E1 catalyze the hydroxylation reaction which is the most important reaction for changing exogenous substances solubility. In addition, CYP2B6, CYP2C8, CYP2C9, CYP2C19, CYP2D6, CYP3A4 and CYP3A5 are known to be expressed in the intestine (Rendic and Guengerich, 2021). CYP3A4 and CYP3A5 are present in the GIT all regions, and CYP3A4 is highly abundant in the duodenum and jejunum (Esteves et al., 2021). CYPs are associated with a variety of metabolic reactions in the body, including oxidation, sulfur oxidation, aromatic hydroxylation, aliphatic hydroxylation, N-dealkylation, O-dealkylation, and deamination. Oxidation, the primary reaction, catalyze non-polar lipid-soluble compounds (containing hydroxyl or aromatic groups) to generate polar oxygen-bound groups.

Additionally, phase I metabolic enzymes that can participate in oxidation reactions includes flavo-protein monooxygenases (FMO), mono-amine oxidase (MAO), diamine oxidase (DAO) and dehydrogenases (e.g., alcohol dehydrogenases (ADHs) and aldehyde dehydrogenases (ALDHs)). FMOs are widely used in the fields of medicine and chemical industry because they often participate in compound hydroxylation, Bayer-Williger oxidation, sulfur oxidation, epoxidation, and halogenation reactions. MAO mainly metabolizes monoamines in organisms, such as adrenergic drugs including 5-hydroxytryptamine (5-HT) (Chen et al., 2015) and catecholamines (Goldstein et al., 2021). Inhibitors of such enzymes are widely used in depression, Parkinson’s and other neurological diseases. DAO is an intracellular enzyme that catalyzes diamines in the mucosa or ciliated epithelial cells of the small intestine (Bounous et al., 1984). It can protect the mucosa by regulating intracellular ion balance and affecting conduction pathways. Other enzymes include nitroreducetase (NRTs), azoreductase (ARTs), esterase, amidase and glucosidase also play an important role in substances metabolism process (Almazroo et al., 2017). Reduction is another important pathway of phase I metabolism, which is important in the metabolism of aromatic nitro, nitroso, azo, and N-oxide compounds. Compounds obtain H from NADH and NADPH to form the corresponding amines. Esterase, amidase and glucosidase are used to hydrolyze ester bonds, amide bonds and glycosidic bonds of lipids, amides and glycosides, respectively, lead to activity reduce or even inactivate.

The principle of using enzymes expressed at targeted sites to engage in bioconversion is a strategy for designing prodrugs. The bioconversion of such new compounds, including but not limited to prodrugs, requires the participation of enzymes, such as esterase (Walther et al., 2017). Prodrugs are cunning derivatives of therapeutic agents designed to improve drug bioavailability. The currently listed prodrugs are mostly ester prodrugs, which need to be activated by esterase hydrolysis of ester bonds. A potent peptidyl inhibitor of aspartic proteases is bioconversion into an open-source antimalarial compound of P. falciparum prodrug activation and resistance esterase (PfPARE), MMV011438 is a good example (Istvan et al., 2017). Another representative prodrug, Prontosil, was reduced to sulfanilamide (SN) with antibacterial activity (Almalki et al., 2022). Another promising new class of drugs, proteolysis-targeting chimera (PROTAC), is also inseparable from the participation of metabolic enzymes (Békés et al., 2022). PROTAC is a bifunctional small molecule bridging a ubiquitin ligase and a target protein. Since the concept was proposed in 2001 (Sakamoto et al., 2001), related industries have developed rapidly. PROTACs has become one of the hotspots in current pharmaceutical research, in which metabolic enzymes play an important role.

### Phase II metabolism pathway

Most of the metabolites produced by the phase I pathway can be excreted directly or after the phase II metabolism, while the other part is directly excreted. Compared to CYP450, phase II enzymes have received relatively less attention in clinical pharmacology, which was outlined in Table 1. The most common phase II drug-metabolizing enzymes are UDP-glucuronosyltransferases (UGTs), sulfotransferases (SULTs), N-acetyltransferases (NATs), glutathione S-transferases (GSTs), methyltransferases (thiopurine S-methyltransferases (TPMTs), catechol O-methyltransferases (COMTs)) and acyltransferases (Jancova et al., 2010; Almazroo et al., 2017). Phase II metabolism reaction is the conjugation, which refers to the binding reaction of the drug or its phase I metabolite with the endogenous substances. The polarity genes of drug molecules are covalently bound with endogenous substances (such as glucuronic acid, sulfuric acid, acetic acid, glycine, *etc.*) to generate highly polar, highly water-soluble conjugates, which are easily excreted in urine and/or bile owing to difficult to reabsorb (Sarlis and Gourgiotis, 2005).

**TABLE 1:** Enzymes involved in phase I and phase II metabolism.

Metabolic types	Reaction types	Enzymes	Cofactor	Reaction	Location
Phase I metabolism	Oxidase systems	CYPs	NADPH + H^+^/O_2_/CYP450	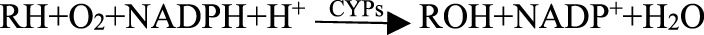	Endoplasmic reticulum (ER)
		FMOs	FAD	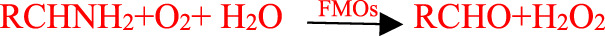	ER
		MAOs	Flavin coenzyme	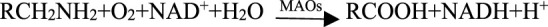	Mitochondria (MIT)
		DAOs	Pyridoxal5-phosphatemonohydrate	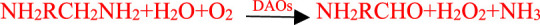	Cytoplasm
		Dehydrogenase (e.g., ADHs, ALDHs)	NAD^+^	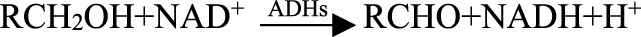	Cytoplasm/MIT
	Reductase systems	NTRs	NADH + H^+^/NADPH + H^+^	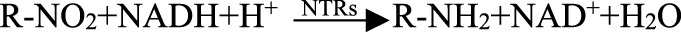	ER
		AZRs		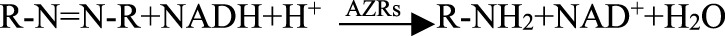	
	Hydrolase systems	Esterase	—	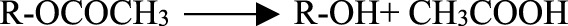	Cytoplasm/ER
		Amidase	—	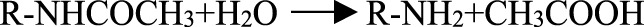	
		Glucosidase	—	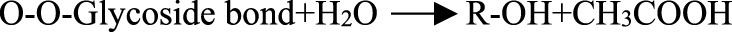	
Phase II metabolism	Conjugated enzyme systems	UGTs	UDPGA	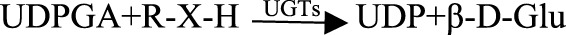	ER
		SULTs	PAPS	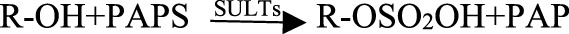	Cytoplasm
		GSTs	GSH	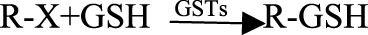	Cytoplasm/ER
	Acylase systems	NATs	Acetyl-CoA	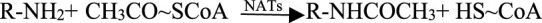	Cytoplasm
		Acyltransferases	Glycinate	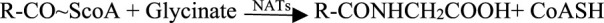	MIT
	Methylase systems	TPMT	SAM	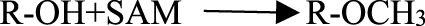	Cytoplasm/ER
		COMT			

UGTs are a drug metabolizing enzymes superfamily that require UDP-glucuronic acid (UDPGA) as a cofactor. The UGT superfamily consists of four families, UGT1, UGT2, UGT3 and UGT8. The glucuronidation reaction catalyzed by the UGTs family, accounting for the phase II metabolism of over 35% clinical drugs (Figure 1C). It catalyzes UDPGA transfer to hydroxyl, carboxyl, or amino groups, resulting in compounds that are more hydrophilic than the substrate (Jancova et al., 2010). Phenobarbiturates combine with GA for corresponding metabolic reactions is a representation (Okada et al., 1969). The current study found that the SULTs are divided into SULT1, SULT2, SULT4 and SULT64 families, of which the SULT1 and SULT2 are participated in more studies (Thelen and Dressman, 2009). The sulfonation reaction mediated by the SULTs family is the primary pathways of phase II metabolism, which participated in various substances detoxification and elimination *in vivo*. The conjugation process catalyzed by SULTs indicated that compounds containing hydroxyl and amino groups to the sulfonic acid group (-SO_3_H) provided by 3′-phosphoadenosine-5′-phosphosulfate (PAPS) (Figure 1D). Sulfonated reaction accounts for a large proportion of exogenous drugs metabolism such as acetaminophen. As far as intestinal phase II drug metabolism is concerned, UGT1A, UGT2B7 and UGT2B15 and SULT1A were shown to be expressed with functional relevance for many drugs, although little quantitative data is available so far (Fritz et al., 2019). GSTs, is the important enzymes family involved in compound metabolism, catalyzing a large number of reactions including nucleophilic aromatic substitutions, Michael additions, hydroperoxides isomeration and reduction, hydrophobic and electrophilic compounds and reduced glutathione conjugation. GSTs are divided into two superfamilies, including the soluble GST superfamily and membrane-associated proteins in eicosanoid and glutathione metabolism (MAPEG, microsomal transferases) (Zarth et al., 2015). Soluble GSTs are subdivided into 8 separate classes designated α, κ, μ, Π, σ, θ, *ζ* and Ω (Salinas and Wong, 1999). The GSTs metabolizing enzyme family is involved in almost all types of drug metabolism. Acetylation is an important amine-containing substances transformation reaction, and acetyl-CoA is a direct donor of acetyl groups. The bioconversion of carboxyl-containing drugs mainly has glycine as a cofactor. The enzyme expression is polymorphic resulting in different acetylation rates among individuals. NATs have been involved in aromatic amines and hydrazines bioconversion by the transfer from acetylcoenzyme A acetyl group to the parent compound free amino group (Sim et al., 2014). NATs are divided into two subfamilies: NAT1 and NAT2, of which NAT1 is expressed in most tissues and mainly affect p-aminobenzoic acid, p-aminosalicylic acid and p-aminoglutamic acid. Meanwhile, NAT2 mainly mediates sulfamethazine, isoniazid, hydralazine and sulfonamide metabolism (Makarova, 2008). Therefore, an appropriate amount of sodium bicarbonate should be supplemented to improve solubility when taking sulfonamides. S-adenosylmethionine (SAM) is an active methyl donor for methylation reactions involving various methyltransferases. TPMT and COMT mediate most of these reactions as the main methyltransferases. TPMTs catalyze the S-methylation of aromatic and heterocyclic sulfur-containing compounds, such as 6-mercaptopurine (6 MP), azathioprine and 6-thioguanine, used in clinical disease treatment (Kouwenberg et al., 2020). COMTs are the phase II enzymes responsible for the transfer of a methyl group from S-adenosylmethionine to its substrate. It is the most efficacious treatment for Parkinson’s disease when combined with decarboxylase inhibitor I-dopa (Espinoza et al., 2012). It plays a key role in the regulation of catechol-dependent functions and metabolism of drugs with catechol functional groups attached to their structures (Volavka et al., 2004).

### Microbiome metabolism pathway

The gut hosts a diverse bacterial community 10-fold larger than human somatic and germ cells, separated from the internal environment by epithelial cells. It has been estimated that the microbes collectively make up to 100 trillion cells, outnumbering host cells. The microbes, encode unique genes, have a profound influence on human physiology (Qin et al., 2010). Gut microbial species include Actinobacteria, Bacteroidetes, Firmicutes, Fusobacteria, Lentisphaerae, Proteobacteria, Synergistetes, Tenericutes, Verrucomicrobia, Ascomycota, Euryarchaeota, Evosea, Fornicata, Fornicata, etc (Guarner and Malagelada, 2003; Adak and Khan, 2019). They are divided into predominant microflora and sub-dominant microflora according to quantity. The quantity of predominant microflora is generally above 10^7^～10^8^ cfu/g, including obligate anaerobic bacteria such as *Bacteroides*, Eubacterium, Bifidobacterium, Rumenococcus and *Clostridium*, which belong to the aboriginal. The amount of sub-dominant microflora is less than 10^7^～10^8^ cfu/g, mainly aerobes or facultative anaerobic bacteria, such as *Escherichia coli* and *Streptococcus* (Javdan et al., 2020). It is unavoidable that drugs spend a significant amount of time in the small and/or large intestines, whether prior to or after absorption. More and more evidence suggest that gut microbiota has both direct and indirect impacts on the metabolism process. The microbiome competes with related metabolic enzymes (Weersma et al., 2020). In addition, the following impact mechanisms are included (Vernocchi et al., 2020): (a) chemical crosstalk between microbial and human metabolic compounds, (b) modulation of immune system, (c) protection from pathogens, (d) enteric nervous system regulation, (e) colorectal cancer resistance, (f) neurological behavior, (g) reduction of lipid levels in serum and cholesterol balancing. Crosstalk refers to the influence of the environment (nutritional, social, behavioral, geographic) on host genetics and the subsequent adaptation of the gut microbiome, triggering the molecular mechanisms of communication between the microbiome and the host. Microbiome self-derived enzymes reflect its direct impact on the bioconversion process. They are mainly involved in the degradation, hydrolysis and reduction through hydrolysis, dehydroxylation, deamidation, decarboxylation and reduction of azide groups (Kararli, 1995). For example, the metabolic pathways of anthraquinones are mainly hydrolysis, glycuronidation, sulfation by intestinal flora and hepatic drug-metabolizing enzymes (Wang et al., 2021). *Bacteroides* species can hydrolyze the steviol glycosides by β-glucosidase (Renwick and Tarka, 2008). This complex metabolic activity recycles valuable energy and absorbable substrates for the host, and also provides energy and nutrients for the flora growth and proliferation (Mancini et al., 2018). Due to the tremendous progress in the study of microbiota structure and function, its contribution to host physiology, metabolism and disease has gradually been understood and appreciated by relevant researchers in recent years (Fung et al., 2017).

## Primary enteric models

The intestine plays a vital role in the absorption of orally ingested compounds, such as nutrients and drugs. Metabolism in the GIT is one of the important determinants of the overall disposition of drugs. However, the significance of the gut in drug metabolic fate has long been underestimated due to the difficulty in distinguishing between the roles of the gut and the liver in *in vivo* experiments and the lack of sufficiently viable *in vitro* models. The good news is that the intestine as an important factor in determining the first-pass metabolic fate of drugs has been increasingly recognized by researchers.

### Microbiome-based model

The role of the microbiota has been largely overlooked previously. Hence the nickname “the forgotten endocrine organ”. The ability of microbes in the human gut to metabolize drugs was discovered nearly a century ago. Orally drugs are exposed to gut flora before being absorbed into the bloodstream. Abundant gut microbes affect compound absorption and metabolism by secreting bioactive molecules such as hydrolase, lyase, oxidoreductase, and transferase to alter drug efficacy and toxicity (Murphy, 2015). The gut microbiome has the ability to produce many kinds of substances. Bacterial culture is often used to study the metabolic effects of intestinal flora on drugs: 1) Frozen glycerol stocks were plated on brain-heart-infusion (BHI) blood agar and incubated at 37°C under anaerobic conditions. 2) Single colonies were inoculated into pre-reduced Gut Microbiota Medium (GMM, 1% w/v arginine). Moreover, gentamicin (200 mg/ml), erythromycin (25 mg/ml), and/or 5-fluoro-2- deoxy-uridine (FUdR) (200 mg/ml) were added. 3) Bacterial incubated anaerobically at 37°C for 24 h (Akkermansia muciniphila for 48 h). Samples were collected and stored at −80°C until further processing for analysis (Zimmermann et al., 2019). Han and his colleagues used isolated gut microbiota in combination with liquid chromatography mass spectrometry to investigate the bioconversion of rare protopanaxadiol saponins. The results showed that ginsenosides Rd, F2 and Rg3 were completely converted *via* deglycosylation. In addition, gut microbiota models for metabolism study are generally developed based on animals (Clarke et al., 2014). Germ-free mice, which do not contain other living organisms, are generally used as controls in experiments. Bäckhed and his co-workers compared the effect of gut microbiota on energy absorption in germ-free mice (C57BL/6) and conventionalized mice, and found that exist of microbiota enhance monosaccharides absorption (Bäckhed et al., 2004). Microbial-based model has many advantages, including the use of affordable and convenient media and can be cultivated on a large scale. In addition, a large number of microbial metabolism studies can be assessed simultaneously. Another key advantage is that higher concentrations of target drugs can be added to microbial cultures compare to animal or enzyme and/or tissue system. Therefore, this facilitates purification and isolation of metabolites and toxicological testing when higher drugs concentrations are used (Lamb et al., 2013). Maria *et al.* developed a gnotobiotic mouse model to separate host and microbiota contributions to drug metabolism (the host-microbiome model) (Zimmermann-Kogadeeva et al., 2020). This model explicitly models gut microbiota activity in the large intestine to identify conditions that promote microbiota contribution to drug metabolism. It is undeniable that a systematic and standardized Microbiome-Derived Metabolism (MDM) map still lacked in contrast to liver-derived metabolism (Javdan et al., 2020). Currently, researchers have been trying to map the MDM of oral drugs using personalized gut microbiome-derived microbial communities (MDM-Screen) to reliably predict and ultimately interfere with the ability of the microbiome to adversely affect drug pharmacokinetics (PK) and pharmacodynamics (PD).

### Cell-based enteric model

**FIGURE 2:**
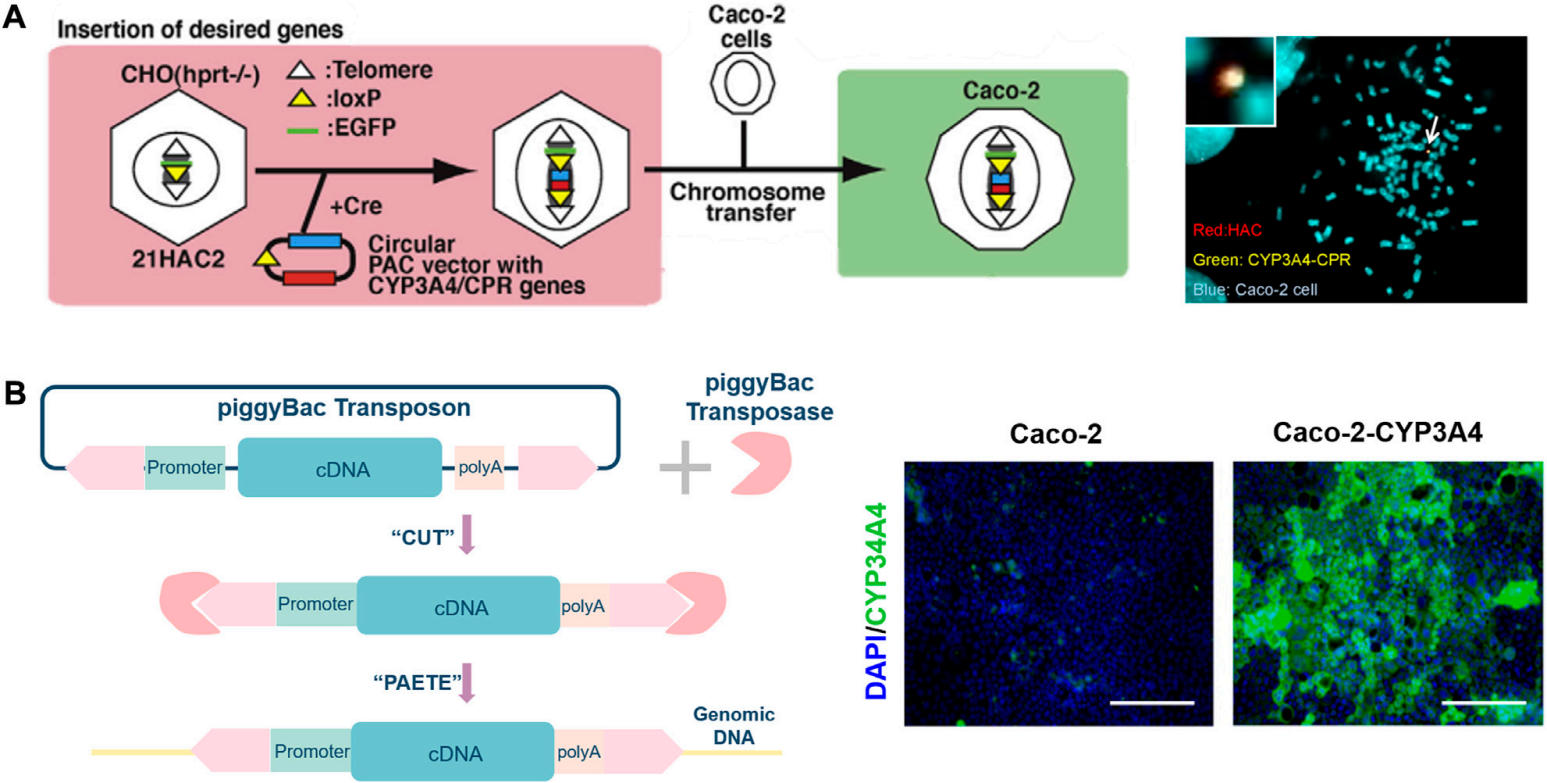
**(A)**. Illustration of construction of the CYP3A4-CPR-HAC and the CYP3A4-CPR-HAC-transferred Caco-2 cells (left), characterization of the CYP3A4-CPR-HAC in Caco-2 cells (right). **(B)**. Schematic illustration of gene editing cell model by the piggyBac transposon system (left), immunostaining analysis of CYP3A4 (green) and nuclei were stained with DAPI (blue) were performed in the Caco-2 cells (Ichikawa et al., 2021). The scale bar represents 200 μm (right).

In the past 10 years, cell culture models have been extensively used to evaluate the behavior of drug candidates and nutrients in the GIT. The Caco-2 cell line model was first proposed (Rousset et al., 1980) in 1980 as *in vitro* human intestinal epithelial cell assay system for predicting gastrointestinal absorption (F_a_) of oral administration. The cell line, is similar to small intestine characteristics, expresses functional P-gp, MRP2/canalicular multispecific organic anion transporter (cMOAT) at levels that allow reproducible absorption and efflux studies in cell culture. Although a gold standard in exogenous substances behavior studies, Caco-2 cell monolayers exhibit certain limitations such as a much higher transepithelial electrical resistance (TEER, up to 500 Ω cm^2^) compared to the human intestine (12–69 Ω cm^2^), overestimated P-gp-mediated efflux and low paracellular permeability, a lack of metabolic mucus and enzymes at their apical side, which limits the relevance of the Caco-2 cell line in metabolism studies under standard culture conditions (Beloqui et al., 2016). CYP3A enzymes, the most abundant P450 present in human hepatocytes and intestinal enterocytes, are heme-containing monooxygenases responsible for the oxidative metabolism of >50% of current drugs on the market (Thummel, 2007). Of the four human CYP3A enzymes identified, CYP3A4 is primarily relevant for drug metabolism, which is involved in the metabolism of approximately 90% of drugs in the gut (van Herwaarden et al., 2009). The discovery of CYP3A4 in the human intestinal mucosa by Watkins and his co-workers and a demonstration that it can operate independently of the liver as a highly efficient metabolic barrier during the uptake of various drugs from the intestine (Watkins et al., 1987; van Herwaarden et al., 2007). The findings of the study illustrate the crucial role that intestinal CYP3A4 expression can have in determining the biological response to an orally dosed substrate. Paine *et al.* found that CYP3A4 is highly expressed in the gut and varies along the length of the small intestine (Paine et al., 1997). To predict the metabolism behavior of drugs in the small intestine accurately, it is necessary to develop a Caco-2 cell model expressing CYP3A4 (Thummel, 2007). Many teams have made various attempts to establish a Caco-2 cell platform that can stably express CYP3A4. Figure 2A clearly demonstrates the use of human artificial chromosome (HAC) vectors to develop Caco-2 cells co-expressing CYP3A4 and CYP450 reductase (CPR) (Takenaka et al., 2017). Specifically, CYP3A4 and CPR genes were cloned into HAC vectors in CHO cells using the Cre-loxP system, and then CYP3A4-CPR-HAC was transferred to Caco-2 cells by chromosomal transfer technology (Ohta et al., 2020). PiggyBac transposon isolated from Trichoplusiani also serves as a tool to overexpress CYP3A4 in Caco-2 cells (Ichikawa et al., 2021). pPB-TRE3G-CYP3A4 and piggyBac transposase vectors were co-transfected into Caco-2 cells and subjected to immunofluorescence analysis (Figure 2). The researchers made various explorations in establishing the Caco-2 cell model expressing CYP3A4. Vector-bearing Caco-2 cells were selected *via* resistance to hygromycin B after cDNAs for CYP450 were introduced into an extrachromosomal vector under the control of the cytomegalovirus early intermediate promoter to develop Caco-2 cell expressing high levels of CYP450 enzymes (Crespi et al., 1996). The treatment of Caco-2 cells with 1α, 25-dihydroxyvitamin D_3_ (1α,25-(OH)_2_-D_3_), beginning at the confluence, results in a dose- and duration-dependent increase in CYP3A4 mRNA and protein (Schmiedlin-Ren et al., 1997). Enhanced CYP3A4 mediated metabolism in Caco-2 cells transduced with Adenovirus-3A4 vector (Ad3A4) and Adenovirus-P450 Reductase (AdRed) (Brimer et al., 2000). Furthermore, there are more and more studies aimed at enhancing the multiple CYP isoforms in Caco-2 cells, including by creating Caco-2 cell lines expressing nuclear receptors (NR) (Korjamo et al., 2006), constitutive androstane receptor (CAR) and pregnane X receptor (PXR) (Burk et al., 2005). With the development of technologies such as gene editing and transfection, researchers can establish models suitable for experiments. Caco-2 cell is highly expressing CYP3A4 can be established and applied to PK studies *via* these technologies.

The researchers need to resort to a new type of intestinal cells to overcome the lack of metabolic enzymes of the Caco-2 cell line was addressed by employing additional cell lines. The TC-7 cell line, one of the Caco-2 cell line subclones, was isolated to overcome the major limitations of the parental line (Ferrec, 2012). There is a good correlation between this subclone and the Caco-2 cell line, indicating that it is an excellent stand-in for Caco-2 monolayers (Grès et al., 1998). Multiple brush border enzymes similar to human enterocyte metabolism, CYP3A4, CYP3A5, UGT and the hydrolase sucrase-isomaltase, were observed on the TC-7 cell line (Liu et al., 2007). Undoubtedly, the original model was eclipsed by the signature of metabolic enzymes that are expressed very similarly to the human empty gut. Consequently, it is reasonable to consider TC-7 cells as a useful option for studying intestine first-pass metabolism.

Stem cells, replenish their own cell population and maintain the potential to develop into more specialized cells, provide an option for metabolism studies to generate large amounts of mature enterocytes/hepatocytes at constant mass (Bacakova et al., 2018). The cells can be divided into two groups based on differentiation potential and origin: (i) adult stem cells derived from host and non-host, such as mesenchymal stem cells (MSC), and (ii) pluripotent stem cells, mainly including human embryonic stem cells (hESC) and human-induced pluripotent stem cells (hiPSC) (Ma, 2014). Jason and his co-workers establish a robust and efficient process to direct the differentiation of hiPSC into intestinal tissue *in vitro* using growth factor manipulations to mimic embryonic intestinal development. The resulting three-dimensional human intestinal organoids (HIOs) consisted of a polarized, columnar epithelium that was patterned into villus-like structures and crypt-like proliferative zones that expressed intestinal stem cell markers (Spence et al., 2011). Janssen *et al.* assess the expression of the most common CYP enzymes in a hiPSC-derived model. This study found relatively high gene expression levels of CYP enzymes in the hiPSC-induced HIO model, indicating that it is a useful *in vitro* gut model for studying chemical bioconversion (Janssen et al., 2021). Yoshida *et al.* establish an *in vitro* differentiation procedure to generate matured small intestinal cells mimicking human small intestine from iPSCs. The tests results confirmed that these iPSC-derived enterocyte-like cells exhibit CYP3A4-mediated metabolism, and can serve as a model for the evaluation of drug metabolism studies in the human small intestine (Yoshida et al., 2021). hiPSC-derived intestinal tissue should allow for unprecedented studies of intestinal wall metabolism.

## Primary hepatic models

Although the study of drugs metabolism in the gut is evolving, it is still falling behind the established liver models to some extent. Most *in vivo* and *in vitro* assessments center around hepatic models nowadays. The liver has long been considered as a principal site responsible due to most of the phase I and phase II metabolism reactions occur with liver metabolic-enzymes participation. It is mainly engaged in physiological processes such as compound metabolism, bile secretion and excretion, detoxification and coagulation factors production. Modern research has found that a number of liver-derived *in vitro* systems, such as slices, primary and immortalized hepatocytes, microsomes and S9 fractions are used to assess the xenobiotics metabolism.

### Hepatocytes model

Nowadays, there are several attempts to establish hepatocyte-based *in vitro* systems as alternatives for animal experiments. The contribution of the liver for oral drugs metabolism is extensively assessed in drug discovery processes by using fresh or cryopreserved hepatocytes and hepatic subcellular fractions. Primary hepatocytes are considered to be a standard *in vitro* tool in the drugs metabolism study (Sahi et al., 2010). Primary hepatocytes include primary human hepatocytes (PHHs) (Zeilinger et al., 2016) and primary mouse hepatocytes (PMHs) (Nagarajan et al., 2019) depending on the species. PHH/PMH is usually isolated from whole livers or resected liver tissue by a continuously modified two-step collagenase perfusion technique, what proposed by Seglen and Reith in 1976 (Seglen and Reith, 1976). The liver tissue separated from the body and rinsed firstly. Then, 5 ml of collagenase V was injected and digested *in situ* for 10 min at room temperature. The liver was then cut into small pieces and placed in 5 ml of collagenase V for further digestion at 37°C for 30 min. Finally, the digested suspension was repeatedly pipetted to detach the hepatocytes, and the tissue debris was washed away with Dulbecco’s modified Eagle’s medium (DMEM) before culture. PHH/PMH have some disadvantages including difficulty in culture, phenotype change at an early stage, metabolic enzymes easy inactivation, and individual differences between donors, which limit their practicability and reliability as an *in vitro* model. Sandwich-cultured hepatocytes (SCHs) is an *in vitro* model widely used in hepatobiliary transport of drugs. Actually, in this establishment of model, hepatocytes are sandwiched between two layers of artificial matrix glues, rebuilding the polarity of cells and forming a complete bile duct network in order to mimic the internal environment of hepatocytes realistically (Fardel et al., 2019). SCHs model expresses drug-metabolizing enzymes especially CYP enzymes, making it valuable to be developed in the application of drug metabolism research (Matsunaga et al., 2016). There is no doubt that SCHs model is deemed as a superior model to traditional PHH. Mardal’s group identify the cannabinoid compound 5F-PY-PICA metabolites *via* the model combined with liquid chromatography-high resolution mass spectrometry/mass spectrometry (LC-HR-MS/MS) (Mardal et al., 2018). In general, co-culture of two or more cells tends to better mimic physiological conditions. HepatoPac is a co-culture model of primary human hepatocytes and mouse fibroblasts that enable long-term hepatic metabolism and toxicity studies (Chan et al., 2019). This technique shows better *in vitro* and *in vivo* correlations than conventional hepatocyte models (Ramsden et al., 2014), especially for medium and low turnover compounds. The architectural organization of HepatoPac cultures has been empirically optimized to promote hepatocyte vitality and enable stable metabolic activity for weeks, rather than hours or days, the typical duration of other culture systems (Kamel et al., 2021).

Hepatic cell lines generated from tumor tissue are widely used in *ex vivo* culture models due to their high proliferation capacity and stable metabolism. Common human hepatic cancer cell lines include HepG2, HepaRG, Huh7, Huh7.5, PLC, Hep3B, SMMC-7721, MHCC97-H, MHCC9-L, *etc.*, each of which has its own characteristics (Fukuyama et al., 2021). SMMC-7721, MHCC97-H, MHCC9-L cell lines have withdrawn from the experimental stage after being confirmed to be contaminated. Human hepatoma cell lines have similar biological properties to primary hepatic cells and unlimited passage, providing an ideal *in vitro* model for cancer and drugs metabolism studies. Therefore, the cell line is used in various fields and is expected by professionals to replace primary hepatocytes. HepG2 and HepaRG, the hepatoma cell lines, are attractive tools for *in vitro* studies under standardized and reproducible conditions (Yokoyama et al., 2018). HepG2 cell line is derived from human hepatoma tissue, which can secrete a variety of plasma proteins. As shown in Table 2, HepG2 cell line is widely used in liver physiology studies (Zhu et al., 2016) with lower levels of specific drug-metabolizing enzymes and transcription factors. HepaRG cell line is a fascinating tool for studying drugs metabolism owing to express liver-specific functions including CYP enzymes, transporters, and nuclear receptors during differentiation (Guillouzo et al., 2007). HepaRG cells express the mature hepatocyte marker, aldolase B, and their mRNA expression level is 20% that of freshly isolated human hepatocytes when highly differentiated into hepatocyte-like cells, while it was not detected in HepG2 cells. The cell line is commonly used in metabolism and toxicity studies due to its high CYP450 enzymes expression (Zanelli et al., 2012). The HepaRG expresses various CYPs (1A2, 2B6, 2C9, 2E1, 3A4), NR, CAR, PXR at levels comparable to PHH and significantly higher than HepG2 (Guillouzo et al., 2007). HepaRG cell are more economical, convenient, and predictable than fresh or cryopreserved PHH (Tascher et al., 2019). The hydroxylation behavior is favored in PHH, whereas the glucuronidation pathway is favored in HepaRG cells. It is available in proliferative state to be expanded and differentiated in-house, or as cryopreserved, fully differentiated and ready-to-use hepatic cells. The Huh7 cell line is used for metabolism and toxicology studies, but also lacks typical hepatic biochemical functions. Malin and his colleagues (Darnell et al., 2012) found that drugs exhibit markedly different bioconversion behaviors in various cellular systems. The long-term differentiated Huh-7 cell line is a promising tool for *in vitro* endogenous compounds hepatotoxicity and metabolism testing. Huh-7 cell line up-regulated some transporters related to farnesoid X receptor (FXR) and nuclear factor erythroid 2-related factor 2 (Nrf2) (Feng et al., 2018; Thomas et al., 2019). These data indicate that it may be used to study drug interactions with MRP when expressed some major drug transporters. Hepatocyte-like cells differentiated from hiPSC-induced cells are of great interest for applications in pharmacological research, especially drug metabolism testing. Murayama team finding that the induction of typical CYP450s in ihPS-derived hepatocyte occurred after normal culture could facilitate the use of these cells for drug metabolism (Murayama and Yamazaki, 2018).

### Hepatocyte subcellular fractions

**FIGURE 3:**
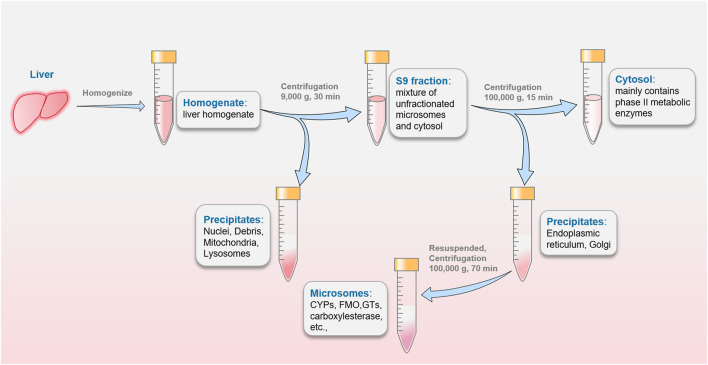
The preparation of S9 fraction, hepatic cytosolic fractions and microsomes commonly used in drugs metabolism studies.

The liver homogenate was subjected to differential centrifugation to obtain subcellular fractions, including hepatic microsomes, S9 fraction and hepatic cytosolic fractions, as showed in Figure 3. Subcellular fractions can be stored and remain stable at −80°C for many years and be thawed easily before experiments. These advantages make sense for studies in the earliest stage of drugs metabolism including the screening program (Parmentier et al., 2007). It should be noted that the corresponding cofactors need to be added to the reaction system to better simulate the physiological environment. S9 fraction is most similar to the physiological properties due to the minimal number of centrifugations. From the macroscopic point of view, S9 fraction is a mixture of unfractionated microsomes and cytosol containing a wide variety of drug-metabolizing enzymes. It is widely used as a preferred test system in several *in vitro* ADME studies including phase I and phase II metabolism (Registre and Proudlock, 2016). S9 fraction has a relatively complete metabolic function to provide a relatively comprehensive metabolic profile, which can better mimic the physiological state (Hamel et al., 2016). Cytosol is the fraction obtained by centrifugation of S9 fraction, and consist of NAT, GST, SULT, *etc.*, enzymes (Neal et al., 2019). This subfraction, mainly contains phase II metabolic enzymes, is used to single soluble enzyme activity and specific metabolic pathways studies (Wahbeh and Christie, 2011). Some exogenous cofactors such as PAPS can be added to stimulate phase II enzymes activity. Hepatic microsomes are mostly existing in ER, so they need to be obtained by differential centrifugation (100,000 g) (Liu et al., 2002). It contains important drug-metabolizing enzymes, such as CYPs, FMO, carboxylesterase and glucuronosyltransferases (GTs), *etc.*, which are responsible for 90% of drug metabolism reactions. Liver microsomes, can be stored for a long time to replace inactivation. It deemed as a vital vector to provide a stable environment relatively for drug metabolism study. Microsome is the most widely used *in vitro* model by far, providing an affordable way to metabolism studies (metabolic profile and prediction of hepatic clearance) and interaction studies (phenotyping and inhibitory potential studies). But exogenous cofactors such as NADPH for CYP and FMO, and UDPGA/alamethicin for UGT need to be added in many experiments. Although it can be easily manipulated in large quantities, it can lead to certain metabolites and metabolic pathways that cannot be determined. Like other *in vitro* metabolic models, liver microsomes not fully mimicking the *in vivo* environment. To alleviate this dilemma, several methods have attracted increasing attention. Formerly, researchers attempted to give the best agreement with *in vivo* clearance values through inclusion of both blood and microsome binding values (Obach, 1999). It is a novel approach to precisely control the reaction by adding different substances to the liver microsomes system. To improve enzyme activity, Liu *et al.* added UDPGA, MgCl_2_, alamethicin, saccharolactone and macelignan to the liver microsomal system (Liu et al., 2014). A specific probe-substrate (cocktail assay) coupled with fast liquid chromatography-tandem mass spectrometry (LC-MS/MS) analysis was developed in human liver microsomes (Zhou et al., 2022). Cocktail assay enables information on multiple metabolic pathways to be obtained in a single experimental procedure with minimal inter-individual effects (Kahma et al., 2021). Furthermore, multiple models are utilized in combination with each other for different experimental purposes (Mohutsky et al., 2006). Nasser and his colleagues analyzed zorifertinib metabolites by using hepatocytes and liver microsomes (Al-Shakliah et al., 2022). Notably, optimal pH in microsomes is important for the physiological interpretation and predictability of intrinsic clearance (CL_int_) (Al-Shakliah et al., 2022).

## Others

### Precision-cut tissue slices

None of above cell cultures, however, can provide a complete intestine or liver model. Precision-cut tissue slices (PCTS) technology refers to cutting fresh tissue into slices of a reproducible and well-defined thickness with a microtome, and incubating with compounds during the experiment (de Kanter et al., 2002). The obtained isolated tissue (intestine or liver) benefit from venous flushing and cooling with ice-cold University of Wisconsin (UW) solution or ice-cold Krebs-Henseleit buffer (KHB) immediately (Palma et al., 2019). It should be noted that KHB should be used simultaneously to prevent intestinal tissue inactivate caused by UW solution single-handed. The tissue microtome (Krumdieck Tissue Slicer) was used for sectioning as soon as possible within 3 h after the tissue was isolated. Practitioners can obtain precision-cut intestinal slices (PCIS) and precision-cut liver slices (PCLS) according to experimental requirements (Groothuis and de Graaf, 2013). Liver slices are typically prepared at a thickness of 250 μm, which allow the inner cell layer to be fully exposed to oxygen and nutrients. Generally, intestinal tissue is suitable for sectioning when filled and/or embedded with low melting point agarose. In order to maintain intestine and/or liver tissues activity, it is necessary to be continuously gassed with 95% O_2_/5% CO_2_. A medium supplemented with glucose and antibiotics (Williams medium E) was used simultaneously. The addition of insulin (30 nM), glucagon (100 nM), corticosterone (1 mM), epidermal growth factor (1 nM) and/or fetal calf serum (5%) may be beneficial for long -term culture (>48 h) (de Graaf et al., 2010; Groothuis and de Graaf, 2013; Palma et al., 2019). The technique is able to preserve drug-metabolizing enzyme and organelle activity and maintain cell-to-cell and cell-matrix interactions (Ioannides, 2013). Moreover, the model can maintain metabolic activity for a long time (8–12 h) and has a stronger environmental tolerance. In recent years, this method has reached the level of precise cutting with the development of slicer technology. To date, it represents a robust and versatile *ex vivo* model without separating cells and keeping the natural cellular environment with a full metabolic program (Othman et al., 2020). The gut is heterogeneous, with distinct structural and functional differences are prominent between duodenum, jejunum, ileum and colon. PCIS is particularly suitable for studying the metabolism of intestine different regions and its effects on the metabolism or transport of drugs (Li et al., 2016). PCLS obtained from rat livers are used in most experiments, other animals including mice, miniature pigs, monkeys and dogs. Human livers have been gradually discovered by researchers in recent years. Where human liver slices can be prepared from small pieces of human liver obtained after partial hepatectomy as surgical waste or as part of non-transplantable donor tissue, this allows for interspecies comparisons and interpretation of human-specific function. Although the expensive price of specially designed tissue slicers limits the model application, its application in scientific research really brings scientist very big technical support. Midwoud and his co-workers integrated PCIS and PCLS obtained from rats into microfluidic chambers to demonstrate gut–liver communication as well as mimicked first pass metabolism by transferring metabolites in PCIS to the PCLS using connected flow (van Midwoud et al., 2010). PCTS from rat and mouse were used in order to figure out the metabolite rate of CYP3A and the formation of 3OH-quinidine in the research (Martignoni et al., 2006). In another case, PCTS was proved as a successful established *ex vivo* model, and was suitable to apply in the drug transport and metabolism testing (van de Kerkhof et al., 2008).

### Isolated tissue perfusion system

Compared with isolated or/and culture cells, it is obviously more reasonable to separate the specific tissue where the metabolic enzymes are located for drugs metabolic research. Relevant practitioners can qualitatively and quantitatively analyze the concentration changes of drug prototypes and their metabolites by using *in vitro* tissue perfusion system (Andlauer et al., 2000). The segment is placed in a bath filled with buffer and perfused with drug after the intestine or liver is removed from an anesthetized animal (Hamed et al., 2021). This method preserves the integrity of tissue structure and function to a certain extent and dynamically monitors the disposal by the intestine or liver, while eliminating other organs interference. Isolated hepatic perfusion is a procedure in which a catheter is placed into the artery to provide blood, and another catheter is placed into the vein to take blood away. This temporarily separates the liver’s blood supply from the rest part circulation, which allows high doses of anticancer drugs to be directed to the target organ. Researchers need to isolate and maintain the tissue at 37°C, then rapidly circulate the perfusate and take samples at specific time points to determine the drug and its metabolites concentration (Windmueller and Spaeth, 1977). Generally speaking, the tissue can basically maintain a normal physiological state under the perfusion state. In order to ensure the activity of drug-metabolizing enzymes, intubation and perfusion oxygen supply should be performed promptly and quickly. Isolated tissue perfusion technique is an effective way to study drugs metabolism and mechanism, but the method requires better perfusion equipment and higher operating technical requirements. The metabolism study of isolated tissue perfusion based on whole organ can exclude the other tissues and organs interference to reflect the state of metabolism truly. The disposition profiles of three of the six major kavalactones (kavain, methysticin and desmethoxyyangonin) and their respective metabolites (p-hydroxykavain, m,p-dihydroxykavain and p-hydroxy-5,6-dehydrokavain) were examined in the perfusate and bile of the isolated perfused rat liver by Fu *et al* (Fu et al., 2012). Based on this, Ma and his colleagues developed a biomimetic and reversibly assembled liver-on-a-chip (3D-LOC) platform and presented a proof of concept for long-term perfusion culture of 3D human HepG2/C3A spheroids (Ma et al., 2018). The model is beneficial for a variety of potential applications, including the development of bioartificial livers, disease modeling, and drug toxicity screening.

### Recombinant enzymes system

With the development of molecular biology, gene recombinant enzymes have been more and more widely used in *in vitro* metabolism study in recent years. Gene recombinant metabolic enzyme is a recombinant enzyme system produced by using genetic engineering and cell engineering to integrate the genes regulating the expression of metabolic enzymes into *E. coli* or insect cells. To facilitate the use of recombinant enzymes to test the substrate specificity *in vitro*, protein arginine N-methyltransferase (PRMT) was cloned in frame into pGEX vectors using standard molecular biology techniques. All nine PRMTs can be expressed in *E. coli* as GST fusion proteins (Cheng et al., 2012). High levels of metabolic enzymes can be expressed in the cell line after culture. The purity of the recombinant enzyme was monitored using SDS-PAGE (Srividya et al., 2016). The recombinant enzyme recovered from the Q-Sepharose anion-exchange column retains full activity for several months if stored at –80°C in the phosphate buffer containing 20% (v/v) glycerol, pH 7.2 (Miziorko and Narasimhan, 2000). After the early stages of purification, recombinases exhibit a significant requirement for stabilizers such as glycerol or substrates. It is an important model for identifying the major metabolic isoenzymes involved in drug metabolism, drug metabolism polymorphisms and drug metabolic interactions. The gene recombinant CYP450 system has a good correlation with the liver microsome experiment, which is suitable for microscopic and detailed research. The model is superior to other *in vitro* methods for specificity and selectivity study of drug-enzyme induction. The gene recombinase system can be used to study compound isoforms mediated by different metabolizing enzyme to produce different metabolites. Asano et al. found that the three metabolites of emetine were epicrine, 9-O-Demethyl Epicrine, and 10-O-Demethyl Epicrine, respectively, by using recombinant P450 enzyme and human liver microsomes *in vitro* incubation method. Among them, CYP3A4 and CYP2D6 catalyze the metabolism of ipecine to epecacine and 9-O-desmethylepecchin (Asano et al., 2001). This method enables to study enzyme structure and function, as well as individual enzymes for substrates and inhibitors. Additionally, it can clarify the results of certain drugs metabolized without the interference of other enzymes. Caroline and others found that CYP2J2 is predominantly expressed in the small intestine and heart *via* this technique and is an unknown player in first-pass metabolism to a certain extent (Lee et al., 2010). The high purity of the expressed metabolic enzymes, specificity and selectivity for different experiments, and an effective means for high-throughput screening and analysis of drugs are the advantages of this technology. However, it has a high application cost and cannot reflect drugs overall metabolism.

### Animal models

No matter how close the *in vitro* models are to the physiological environment, the progress of biomedical research still relies on animal models as the experimental basis for experimental and clinical hypotheses. It is readily appreciated that the information obtained from *in vitro* experimental systems is limited. The advances in detection technology and equipment have made major strides that are in charge of making animal models with more and more reliable predictability. There are three types of animal models: homologous (identical to humans), isomorphic (resembling a human disorder) and predictive (allowing the prediction of human disease and treatment) (Xu et al., 2021). The animal models employed to studying the behavior of drugs are rats, mice, rabbits, pigs, canines and sheep, of which rats and mice are the most commonly used species (Shrestha and Préat, 2020). It must be mentioned that the gut microflora, higher metabolic activity and fecal reabsorption are differing from humans though the GIT barrier similar to human beings. An appropriate animal model should be selected on the basis of study purpose, such as transport proteins and metabolic enzymes expression. Indeed, no species is identical to humans at the functional level for any metabolize enzyme, but more similarities are found in higher species. Recent research data provide novel evidence on these observed similarities and differences by molecular biology methods (Tang and Prueksaritanont, 2010). Non-human primates (rhesus (Twaddle et al., 2019) and cynomolgus monkeys (Shen et al., 2021)) have metabolic similarities to human especially for CYPs (Uno et al., 2018), and the chimpanzee has been characterized as a surrogate for drug oxidation and glucuronidation in humans and as a PK model for the selection of drug candidates (Yang et al., 2014). Interestingly, pigs can also be a good model for studying active compounds mainly metabolized by aldehyde oxidase (AOX1), NAT (NAT1 and NAT2) or cytochrome (CYP2C9-like) enzymes (Dalgaard, 2015). Bioanalytical technologies (liquid chromatography (Hsieh and Korfmacher, 2006), mass spectrometer (Hsieh, 2008), *etc.*) are very common methods for evaluating drugs metabolism by collecting blood, bile, urine, feces and tissue samples after administration (Dunn et al., 2011; Chen and van Breemen, 2020). The method can reflect and even quantify the metabolic results of drugs *in vivo* on a macroscopic scale, but cannot accurately judge the effects of various parts. Obviously, the prerequisite for a valid animal model is similarities to humans in terms of target orthologous CYPs, substrate specificity, response to the inhibitor and disposition mechanism.

**FIGURE 4:**
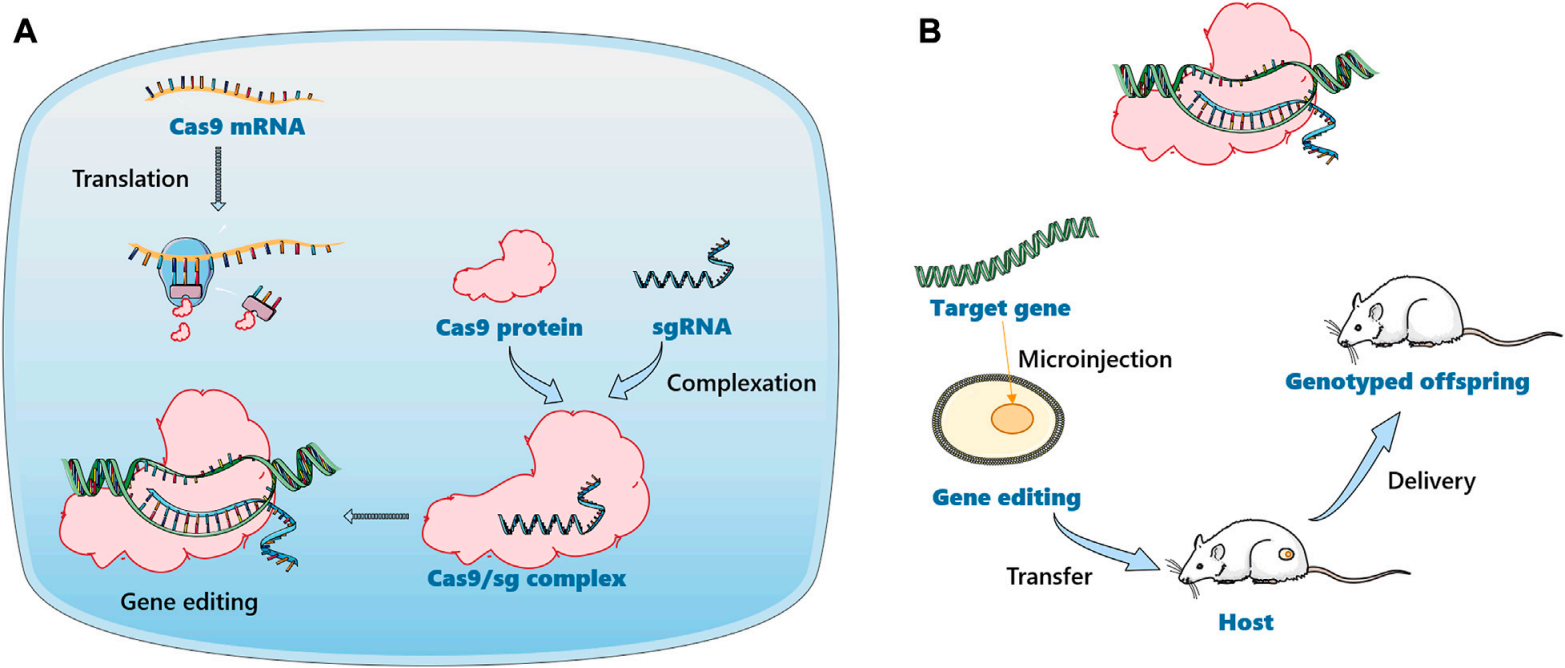
Schematic illustration of gene editing animal model construction based on CRISPR-Cas9 technology. **(A)**. The delivery of a mixture of Cas9 mRNA and sgRNA. Cas9 mRNA will be translated into Cas9 protein in the cell to form the Cas9/sgRNA complex. **(B)**. The Cas9/sgRNA complex was delivered directly into the cell.

With the development of molecular biology and genetic engineering technologies, humanized animal models such as transgenic mice, gene knockout mice and chimeric mice have appeared one after another (Bandzar et al., 2013; Baker et al., 2019). More advanced gene editing technologies, such as zinc-finger nucleases (ZFNs) (Geurts et al., 2009), transcription activator-like effector nucleases (TALENs) (Mak et al., 2012) and clustered regularly interspaced short palindromic repeats-CRISPR-associated (CRISPR/Cas9) (Hendriks et al., 2021) were developed, which were used for gene knock-in and knock-out in animals to construct gene editing animal models. ZFN technology consists of the DNA binding domain of zinc-finger protein and the DNA cleavage domain of Fok I endonuclease, while TALENs consist of transcriptional activator-like effector (TALE) protein and DNA cleavage domain. ZFN technology requires highly skilled experts and screening of ZFN libraries to design, while TALENs have disadvantages such as large size, prokaryotic origin, and cytotoxicity. Compared with other gene-editing technologies, CRISPR-Cas9 technology is simple, efficient, and very specific (Zhang et al., 2019). The technology involves two key components: a single guide RNA (sgRNA) matching the target gene and Cas9 protein causing double strand DNA break (Lu et al., 2021). Various modifications can be performed in the CRISPR-Cas9 cargo system, as shown in Figure 4, i.e., plasmid DNA encoding sgRNA and Cas9, the combination of sgRNA and Cas9 mRNA, and the combination of sgRNA and Cas9 protein (Sharma et al., 2021). CRISPR-Cas9 technology has developed into a general tool for genome editing, especially for generating robust animal models. An increasing number of engineered mouse/rat models are being used to study the effects of metabolizing enzymes, and the target genes are mainly concentrated in the CYP450 family (Karlgren et al., 2018; Li et al., 2019). Since the first report about CYP450 knockout mouse appeared in 1995, numerous of CYP-knockout and CYP-cDNA transgenic mouse model were created for experimental need (Pineau et al., 1995). So far, CYP-knockout mice models have served to drug metabolism studies, especially in CYP gene family 1–4 (Wei et al., 2013). The role of different kinds of CYP450 enzymes have been researched in the metabolism of acetaminophen (APAP), commonly known for hepatotoxicity, in the examples of CYP450 knockout mouse model (Zaher et al., 1998). Abedelmegeed and his companions found that APAP primed liver damage and protein adduct formation was inhibited in CYP2E1 knockout mouse as well as CYP1A2/CYP2E1 double knockout mouse (Abdelmegeed et al., 2010). Nevertheless, APAP bought the risk of hepatotoxic and death of CYP1A2 knockout mouse in comparison to wild type mice, which indicated the involvement of CYP1A2 was minimal compared to the involvement of CYP2E1. In addition, the application of CYP-knockout mouse model participates in chemical carcinogens. Of note, it is recognized that the initial bioactivation of P450 or other bioconversion enzymes is the vital step. Thus, different types of CYP-knockout mouse model vary protective or damaging outcomes after chemical carcinogens metabolism. 3-methylindole, a lung and nasal chemical carcinogens in tobacco smoke, were used in CYP2A5 knockout mouse and CYP2F2 knockout mouse, respectively. Zhou *et al.* demonstrated that although both of them metabolized 3-methylindole *via* either epoxidation or dehydrogenation pathways, CYP2F2 was favorable to produce reactive iminium ions while CYP2A5 was favorable to produce stable derivatives, which caused different degrees of injury to mouse (Zhou et al., 2012). There are numerous factors that affect the accuracy of the test in the long and complicated operation process, including individual differences. Therefore, it is best to combine with other *in vitro* models to accurately reflect the real situation of drugs *in vivo*.

## Correlations between *in vitro* and *in vivo* studies

Increasing emphasis is being placed on using *in vitro* models results for drugs as a surrogate for their *in vivo* behavior. *In vitro* to *in vivo* extrapolation (IVIVE) can convert *in vitro* drug metabolism data into *in vivo* metabolism data (Algharably et al., 2022). During drug development, a variety of *in vitro* metabolic models are utilized to screen and study the metabolic properties of candidate compounds. Based on relevant data, researchers modify drugs to improve metabolic stability and bioavailability. The results of *in vitro* experiments are generally served for *in vivo* experiments, and two types of data need to be combined before used. The *in vivo* drug clearance rate derived *in vitro* is often lower than the *in vivo* measured value within a three- to 10-fold error range (Bowman and Benet, 2019). Researchers established *in vitro* metabolic data to infer *in vivo* metabolic models to overcome this dilemma. It is common practice to measure the CL_int_ of drugs *in vitro* using microsomes or hepatocytes to predict the *in vivo* CL (Lam and Benet, 2004; Sohlenius-Sternbeck et al., 2012). Afterwards, the correction equation of hepatic CL_int_ was established for *in vitro* metabolism experiments (Poulin and Haddad, 2013).

Intrinsic CL:

$${CL}_{int\left(in\;vivo\right)}={CL}_{int\left(in\;vitro\right)}\times SF$$ (1)

Conventional:

$$CL=\frac{Q_{liver}\times f_{u_{b}}\times {CL}_{int\left(in\;vivo\right)}}{Q_{liver}+f_{u_{b}}\times {CL}_{int\left(in\;vivo\right)}}$$ (2)

Conventional bias corrected:

$$CL=\frac{Q_{liver}\times f_{u_{b}}\times {CL}_{int\left(in\;vivo\right)}}{Q_{liver}+f_{u_{b}}\times {CL}_{int\left(in\;vivo\right)}}\times \frac{1}{{AFE}_{conventional\left(method\right)}}$$ (3)

Berezhkovskiy:

$$CL=\frac{Q_{liver}\times f_{u_{b-app}}\times {CL}_{int\left(in\;vivo\right)}/f\_\left(u\_inc\right)}{Q_{liver}+f_{u_{b-app}}\times {CL}_{int\left(in\;vivo\right)}/f\_\left(u\_inc\right)}$$ (4)

Poulin:

$$CL=\frac{Q_{liver}\times f_{u_{liver}}\times {CL}_{int\left(in\;vivo\right)}/f\_\left(u\_inc\right)}{Q_{liver}+f_{u_{liver}}\times {CL}_{int\left(in\;vivo\right)}/f\_\left(u\_inc\right)}$$ (5)

Direct scaling:

$$CL=\frac{Q_{liver}\times {CL}_{int\left(in\;vivo\right)}}{Q_{liver}+{CL}_{int\left(in\;vivo\right)}}$$ (6)

Regression equation:

$$CL=\frac{Q_{liver}\times {CL}_{int\left(in\;vivo\right)}}{Q_{liver}+{CL}_{int\left(in\;vivo\right)}}\times \log{CL}_{int\left(in\;vivo\right)}=0.66\times \log\left[{CL}_{int\left(in\;vitro\right)}\times SF\times \frac{f_{u_{b}}}{f\_\left(u\_inc\right)}\right]+0.633$$ (7)

where CL_int_ and Q represent the intrinsic clearance and the liver blood flow rate, respectively. fu_b_, fu_b-app_ and fu_liver_ are the unbound fraction in blood, apparent unbound fraction considering the pH gradient and unbound fraction in liver considering the protein-facilitated uptake and pH gradient. fu_inc_ is the unbound fraction in incubation medium (hepatocytes). SF is the scaling factor (i.e., the physiological SF was (99 × 10^6^ cells/g liver) × (1799 g liver/70 kg body weight). AFE obtained from the conventional method predictions for each dataset studied; therefore, a different AFE value was used for each dataset. In regression equation, 0.633 represent the intercept and 0.670 is the slope.

In general, liver microsomes have higher CL_int_ than hepatocytes. Therefore, in the absence of significant active uptake transporter action, liver microsomes are more accurate (Bowman and Benet, 2019). These are of great significance to help predict the value of drug development in advance, provide guidance for *in vivo* experiments, and save development time and costs (Jones et al., 2022).

Physiologically-based pharmacokinetic (PBPK) and metabolism patterns are used to predict and explain the differences in ADME properties of drugs among individuals, which are crucial for simplifying drug formulation development and regulatory evaluation (Chow and Pang, 2013). Currently, PBPK models are usually utilized as a prominent tool to use *in silico* (distribution) and CL and provide a predictable process of the overall PK profile, which applies in the pharmaceutical industry constantly (Kostewicz et al., 2014). PBPK integrates information such as system properties, drug properties, formulation properties, and anatomical structures of tissues or organs through mathematical models, which can provide a comprehensive description of the *in vivo* PK behavior of drugs (Dubaj et al., 2022). The model can accurately reflect the drug concentration vs time *in vivo*, as well as the effects of disease and physiological factors on PK behavior. ADME properties of drugs cleared primarily by the liver within *in vivo* systems were determined according to PBPK modeling. Moreover, results of *in vitro* tests have to be related back to the biological context where metabolism and redistribution occurs, which can be accomplished in part using IVIVE method (Hines et al., 2022). Thus, PBPK modeling and IVIVE are indispensable tools for drug development and interpretation. Compartment model, an abstract concept that does not necessarily represent a specific anatomical part, is the basic analytical method used in PK (Nestorov et al., 1998). The one-compartment model means treating the host as a kinetic unit, which is appropriate for situations where the instantaneous distribution of drugs reaches a dynamic equilibrium. According to the different transport rates exhibited by drugs between different parts *in vivo*, the part of a rich blood supply and a higher transport rate is called the central compartment. Others are called peripheral chambers, and are further divided into primary peripheral chambers, second peripheral chambers, *etc.*, which are called multi-compartment models. In addition, there are non-compartmental models that cannot be defined by existing information (Jaki and Wolfsegger, 2012). While no single method emerged as superior of all the compounds evaluated, multiple approaches exhibit a higher value. For drugs with low pharmacological activity, the differences between compartments can be ignored. For targeted drugs, the classical compartment model cannot be applied. Notably, a drug can be described by different compartment models with significantly different parameters.

## Conclusion and future work

**FIGURE 5:**
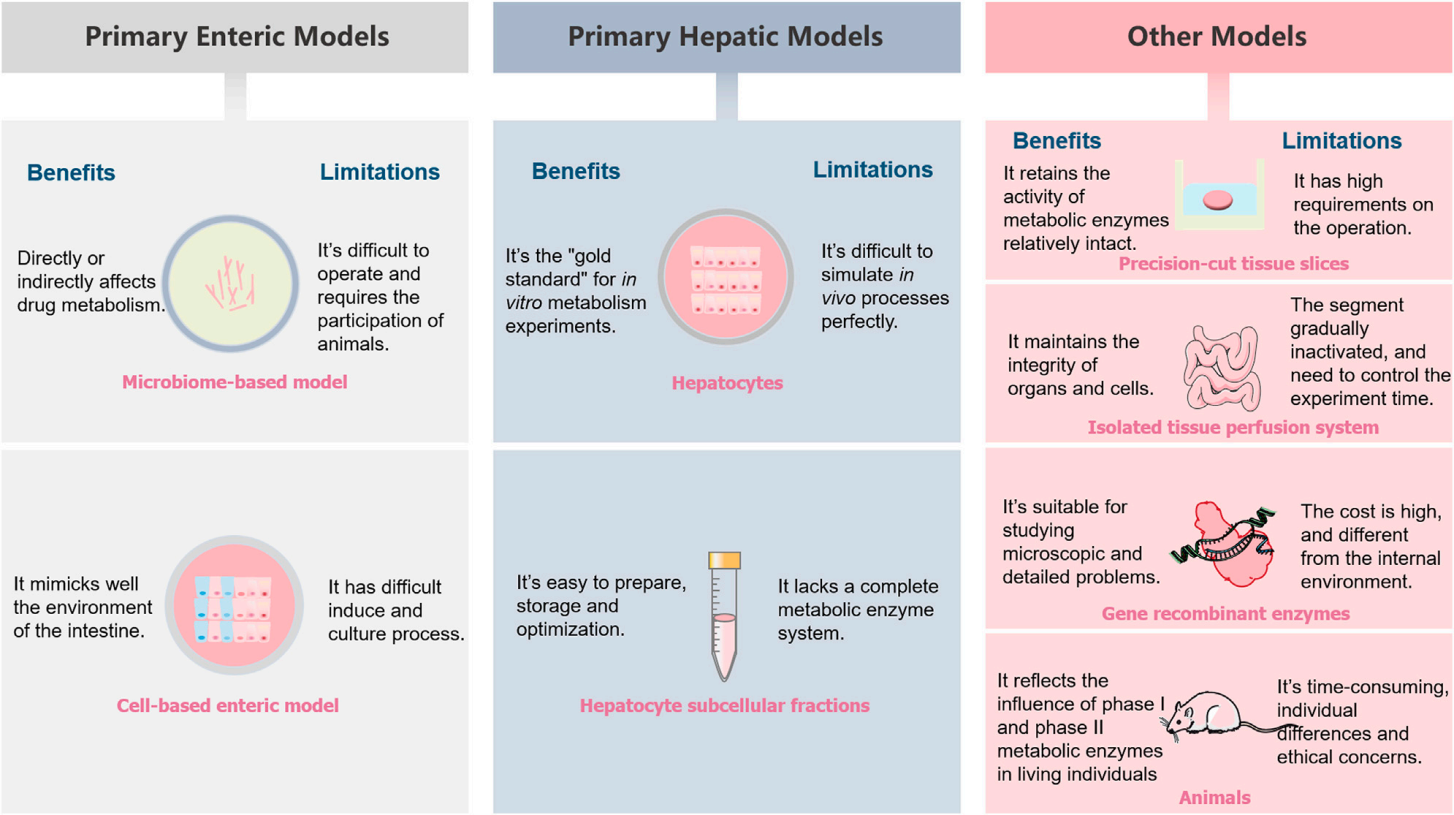
A schematic illustrations summarizing the benefits and limitations of different metabolism models.

For scientists in specialized fields, exploring the metabolic processes and mechanisms of drugs *in vivo* is a significant and ongoing clinical challenge. Drug discovery and development are a costly and often time-consuming activity. A tremendous amount of research effort has been devoted to using various models to evaluate the metabolic fate of drugs *in vivo*. A variety of drug metabolism models are presented in the article, their inherent deficiencies lead to a limited extent and accuracy of prediction in clinical studies though these models are of great significance for drug development. The benefits and limitations of these models are shown in Table 3 and Figure 5. Researchers should choose models and experimental techniques that have been validated by extensive experiments, which are of great value in improving the data accuracy. To date, *in vivo* models are still the important models which able to predict drugs metabolic fate. For *in vitro* models, the classic cell culture model remains the most widely used tool for many years due to its low cost and ease of use. These test systems are preferably *in vitro* models, aiming at reducing the use of animals. However, take into account the relative impact of many biological parameters on the metabolic fate of drugs in the human body, sophisticated models are necessary. Researchers should focus on improving existing models and/or creating new biomimetic models to improve model accuracy. The emergence of *in silico* models has promoted the development of drug metabolism research techniques. *In silico* models have become an ideal aid for the evaluation of metabolic experiments due to their rapidity and high throughput. *In silico* models related to metabolism mainly include: 1) metabolism enzyme substrate and inhibitor classification model, 2) metabolic site prediction model, 3) metabolite prediction model, 4) liver clearance prediction model. Considering the importance of the model in studying drugs metabolism, further study of it is also of great significance for the development and improvement of future metabolic models. Only predominate the sticking point information about the drugs metabolism can researchers choose the appropriate experimental method according to their need. Undoubtedly, this will improve the accuracy of experiments as well as reduce costs, both in time and money. In order to develop more effective clinical drugs, robust and representative model combination is urgently needed. It helps to reduce the high elimination rate in the drugs research and development process, and lays the foundation for the smooth launch of new drugs.

**TABLE 2:** Summary of commonly used hepatoma cell lines.

Cell line	Metabolic enzymes	Application	References
PHH	Complete hepatocyte metabolic enzyme system	*In vitro* drug metabolism studies	Zeilinger et al. (2016)
HepG2	Lower levels of liver-specific drug-metabolizing enzymes and transcription factors	Hepatocyte metabolism studies	Zhu et al. (2016)
HepaRG	CYP1A2, CYP2B6, CYP2C9, CYP2E1, CYP3A4, NR, CAR and PXR	*In vitro* drug metabolism studies	Zanelli et al. (2012)
Huh7	Produces some cytoplasmic proteins, but lacks some metabolic enzymes	*In vitro* hepatotoxicity and endogenous compound metabolism studies	Feng et al. (2018)

**TABLE 3:** The benefits and limitations of different metabolism models.

Models		Benefits	Limitations
Primary enteric models	Microbiome-based model	Directly or indirectly affects drug metabolism	It is difficult to operate and requires the participation of animals
	Cell-based enteric model	It mimicks well the environment of the intestine	It has difficult induce and culture process
Primary Hepatic Models	Hepatocytes	It is currently the “gold standard” for *in vitro* drugs and xenobiotics metabolism experiments	It is difficult to simulate *in vivo* processes perfectly
	Hepatocyte subcellular fractions	It is convenient and easy to prepare, has good reproducibility, easy storage of enzyme mixture and easy optimization of incubation conditions	It lacks a complete metabolic enzyme system
Others	PCTS	It retains the activity of metabolic enzymes relatively intact, allowing the simultaneous analysis of multiple metabolites	It has high requirements on the operation of the experimenter
	Isolated tissue perfusion system	It maintains the integrity of organs and cells, allowing experiments to be performed under near-physiological conditions	It takes a long time, and the segment tends to lose activity
	Gene recombinant enzymes	It is suitable for studying microscopic and detailed problems in the field of metabolism	The experiment cost is high, and the conditions are quite different from the internal environment
	Animals	It reflects the influence of phase I and phase II metabolic enzymes in living individuals	The experimental process is expensive, time-consuming, difficult to operate, has individual differences and ethical concerns
